# In This Issue: Volume 116, Issue 12, December 2025

**DOI:** 10.1111/cas.70268

**Published:** 2025-12-01

## Liposomal ^188^Rhenium Plus Macrophage Depletion Enhances Anti‐PD‐L1 Efficacy and B Cell Infiltration Against Lung Metastatic Cancer

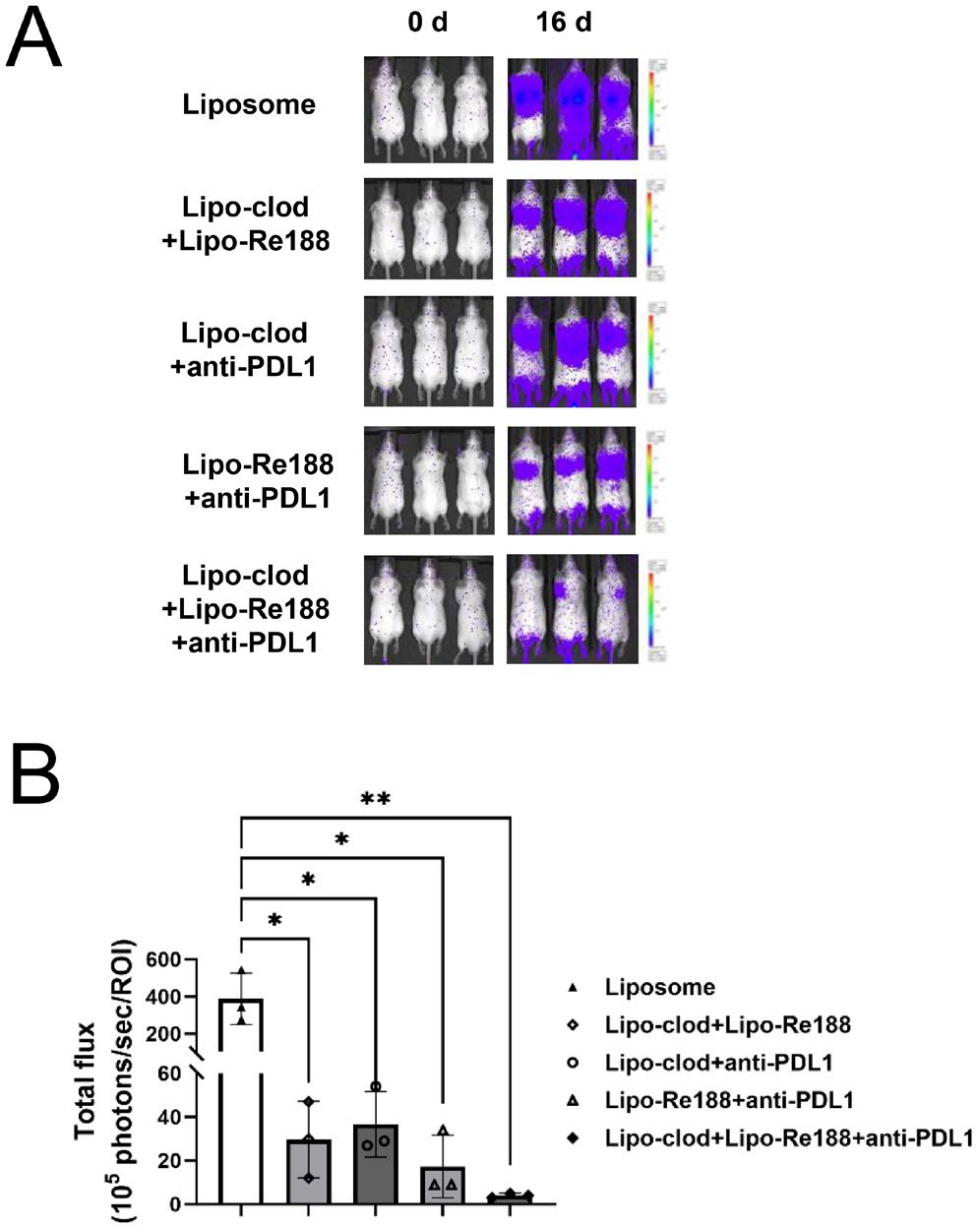

Radionuclides, or radioactive forms of an element, represent a promising therapy for metastatic cancer, as their ionizing radiation can effectively target, kill cancer cells, and limit damage to nearby tissues. Consequently, liposome‐encapsulated radionuclides have garnered significant attention, as liposomes' lipid bilayer protects radionuclides and prolongs their circulation time, while their surface can be modified with antibodies for targeted delivery.

One such promising radionuclide is rhenium‐188 (Re‐188), which has shown the ability to trigger an anti‐tumor immune response. However, the body's mononuclear phagocyte system (MPS)—consisting of liver Kupffer cells, splenic macrophages, and bone marrow phagocytes—presents a significant obstacle as it rapidly removes liposomes from the bloodstream. *Additionally*, when radionuclides decay inside MPS macrophages, they may impair these cells' phagocytic function and disrupt immune balance, possibly worsening immune toxicity with repeated doses.

To address this issue, Liu et al. tried to improve the therapeutic effect of liposome‐encapsulated Re188 (Lipo‐Re188) in lung cancer through macrophage depletion and immune checkpoint blockade.

For this, the researchers established a lung metastatic colon cancer mouse model and assessed the impact of a triple combination therapy consisting of Lipo‐Re188, liposomal clodronate (Lipo‐clod), and anti‐PD‐L1 antibody. They hypothesized that Lipo‐clod‐mediated macrophage depletion would reduce MPS clearance of Lipo‐Re188, thereby enhancing its therapeutic efficacy. The anti‐PD‐L1 antibody was included because Lipo‐clod treatment increases circulating neutrophils, which express high levels of PD‐L1 that can suppress anti‐tumor immunity.

The findings demonstrated that the triple combination therapy effectively remodeled the tumor microenvironment through several mechanisms. Macrophage depletion enhanced Lipo‐Re188 tumor accumulation, promoted B cell recruitment, and reduced interstitial macrophages. Meanwhile, PD‐L1 blockade counteracted the compensatory PD‐L1 upregulation induced by Lipo‐clod/Lipo‐Re188. These changes collectively led to sustained increases in B and T cell populations, creating a more immunostimulatory and tumor‐suppressive environment.

Using this proof‐of‐concept, researchers may be able to develop new combinations of radiation therapy and checkpoint inhibitor therapy for more effective treatments against cancer.

https://onlinelibrary.wiley.com/doi/full/10.1111/cas.70206

## Near‐Infrared Photoimmunotherapy Targeting Esophagogastric Junction Adenocarcinoma with Fully Human Anti‐EpCAM Antibody

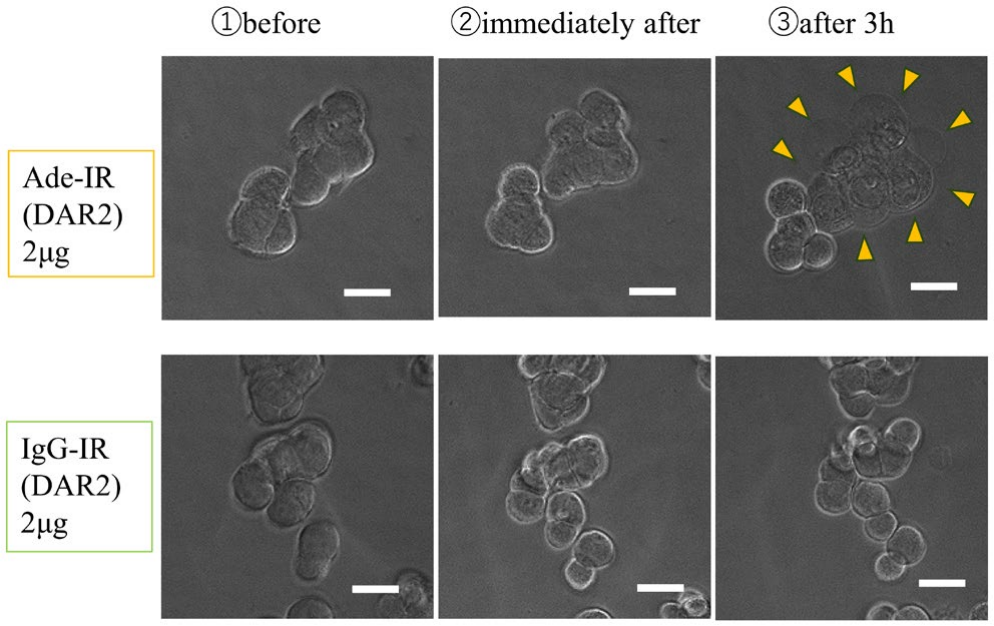

Cancers that originate at the point where the esophagus meets the stomach, called esophagogastric junction (EGJ) cancers, are becoming more common worldwide. These cancers are difficult to treat. Surgery and chemotherapy can help, but they often harm healthy cells and do not always prevent the cancer from recurring.

This study introduces a new treatment approach called Near‐Infrared Photoimmunotherapy (NIR‐PIT). It uses a specialized light source to activate a medicine that is delivered directly to cancer cells. The medicine contains an antibody—a protein that can recognize and bind to cancer cells—combined with a light‐sensitive dye. When near‐infrared light is directed onto the tumor, the dye activates and selectively destroys only the targeted cancer cells, while leaving healthy cells unharmed.

To identify the best target for this therapy, the researchers studied tumor samples from 46 patients and examined three possible candidate molecules: epidermal growth factor receptor (EGFR), human epidermal growth factor receptor 2 (HER2), and epithelial cell adhesion molecule (EpCAM). They found that EpCAM was present in nearly all samples and was distributed uniformly throughout the cancer tissue.

Based on this discovery, they created a treatment using a fully human antibody called adecatumumab, which binds to EpCAM and carries the light‐sensitive dye. Tests in cell cultures and in mice showed that, after light exposure, this therapy successfully destroyed tumor cells and significantly prolonged survival. The results suggest that NIR‐PIT targeting EpCAM could provide a highly specific and less harmful treatment option for EGJ cancer, reducing the limitations of conventional therapies. Since the antibody is made from human proteins, it may be suitable for future clinical use.

This research offers new hope for people with EGJ cancer by combining innovative science and precision targeting with light‐activated therapeutic approaches to fight cancer more safely and effectively.

https://onlinelibrary.wiley.com/doi/full/10.1111/cas.70186

## Reprimo (RPRM): A Tumor Suppressor that Induces Extrinsic Apoptosis via YAP Signaling

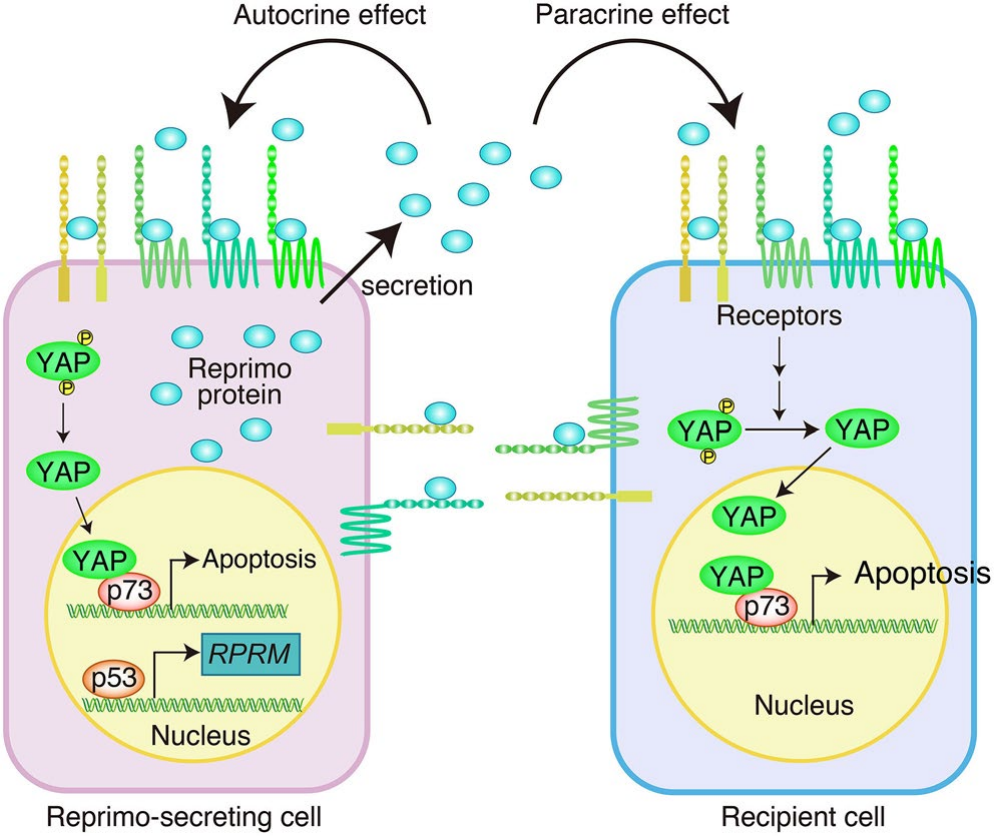

Reprimo (*RPRM*) is a tumor suppressor protein originally identified as a target of the p53 gene, one of the key regulators of cell growth and DNA damage response. Reprimo helps maintain cellular integrity by halting the cell cycle and inducing apoptosis, a form of programmed cell death that removes damaged cells before they become cancerous. However, in many types of cancer, including those of the stomach, breast, and pancreas, the *RPRM* gene is silenced through promoter hypermethylation, allowing abnormal cells to escape control and proliferate unchecked.

In this issue, Takikawa and Ohki review recent discoveries revealing that Reprimo functions not only within cells but also outside them. They describe how secreted Reprimo acts as an extracellular messenger that binds to specific receptors on neighboring cells. This binding triggers a signaling cascade involving protocadherin family proteins and the Hippo–YAP pathway, ultimately leading to apoptosis in target cells. Importantly, this mechanism selectively eliminates cancer cells while sparing healthy ones, highlighting a previously unrecognized tumor‐suppressive role of Reprimo in the extracellular environment.

The authors further discuss how Reprimo represents the first known extracellular ligand capable of inducing cell death through YAP signaling, positioning it as a unique upstream regulator of the Hippo pathway. Loss or suppression of Reprimo expression may therefore contribute to tumor growth and metastasis. Understanding this pathway opens possibilities for therapeutic strategies that reactivate or mimic Reprimo function. Such approaches could promote natural cancer cell death, improve early detection, and enhance the precision and safety of future anticancer treatments.

https://onlinelibrary.wiley.com/doi/full/10.1111/cas.70215

